# A new *Antaeotricha* species from Southeastern Arizona (Gelechioidea, Elachistidae, Stenomatinae)

**DOI:** 10.3897/zookeys.57.462

**Published:** 2010-09-21

**Authors:** Clifford D. Ferris

**Affiliations:** 5405 Bill Nye Ave., R.R. 3, Laramie, WY 82070, USA. Research Associate: McGuire Center for Lepidoptera and Biodiversity, Florida Museum of Natural History, University of Florida, Gainesville, FL; C. P. Gillette Museum of Arthropod Diversity, Colorado StateUniversity, Ft. Collins, CO; Florida State Collection of Arthropods, Gainesville, FL

**Keywords:** Antaeotricha arizonensis, Arizona, Elachistidae, Gelechioidea, New Mexico, Stenomatinae

## Abstract

The new species Antaeotricha arizonensis is described from southeastern Arizona and southwestern New Mexico. Adults and genitalia are illustrated.

## Introduction

Duckworth (1964) reviewed the North American Stenomidae (now treated as Stenomatinae based on the revision by Hodges 1999, p. 136) and described only two new taxa: Antaeotricha fuscorectangulata (TL South Fork of Cave Creek, Chiricahua Mts., [Cochise Co.] Arizona, and Mothonica kimballi (TL Siesta Key, Sarasota County, Florida). In 1968, Jerry Powell and Paul Opler reared an Antaeotricha species from southeastern Arizona from larvae found in Madera Canyon, Santa Rita Mts., and in 1988 Powell reared specimens from Miller Canyon, Huachuca Mts. The moth was not described. Since 2004 I have collected specimens of this same species in UV light traps in the Chiricahua and Mule Mts. in Cochise Co., Arizona, and in the Pinos Altos Mts., Grant Co., New Mexico. Antaeotricha arizonensis is now described from a series of ninety-three specimens.

## Taxonomy

### Antaeotricha arizonensis

Ferris sp. n.

urn:lsid:zoobank.org:act:521FAB65-4310-42E2-83C8-41099160AC46

Figs. 1–9 

#### Type material.

**Figures 1–9.**
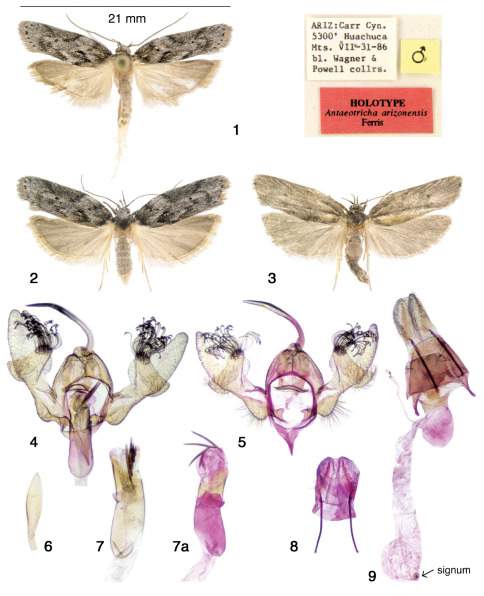
Antaeotricha arizonensis adults and genitalia. **1** Male holotype and pin labels **2** Fresh adult female, AZ Cochise Co., Pinery Canyon, 19.viii.04 **3** Worn adult female, AZ Cochise Co., Shaw Peak Trail–Onion Saddle, 10.x.07 **4** Male genitalic capsule **5** Male genitalic capsule with aedeagus removed (see 7a) **6** uncus tip flattened **7** Aedeagus (corresponding genitalic capsule not shown), (7a) with vesica partially everted (from genitalic capsule in Fig. 5) **8** Flattened ovipositor lobes of female genitalia **9** Complete female genitalia.

**Holotype** male (Fig. 1): **Arizona**, Cochise Co., Huachuca Mts., Carr Canyon, 5300’ (1617m), 31.vii.1986, Wagner & Powell; deposited in the Essig Museum of Entomology (EME), University of California, Berkeley, CA. **Paratypes** (deposited in EME): **Arizona**, Pima Co. Larvae Santa Rita Mts., Madera Canyon, 5–6.vi.1968, emerged 22.vii.1968 (1m, 1f), P. Opler, reared from Quercus hypoleucoides under lot no. J. Powell 68F54-55. Cochise Co. Larvae Huachuca Mts., Miller Canyon, 1775m, 14.iv.1988, emerged 28.vi–3.vii.1988 (2m, 2f), J. Powell, reared from Quercus hypoleucoides under lot no. J. Powell 88D34.; Huachuca Mts., Miller Canyon, 5800’ (1617m), 3.viii.1986 (1m, 2f) at black light, J. W. Brown & Powell; 7.viii.1991 (1f), J. A. Powell; (in author’s collection): **Arizona**, Cochise Co., Chiricahua Mts., above Onion Saddle, 7630’, 22.vii.2007 (1f), 10.x.2007 (1f); Pinery Canyon, 7000’, 19.viii.2004 (1m), 15.viii.2004 (5m, 2f), 24.viii.2006(2m); Mule Mts., 5700’, Banning Creek, 16.vi.2009 (1m). **New Mexico**, Grant Co., Pinos Altos Mts., 7975’, Signal Peak, 23 August, 2004 (1m); all C. D. Ferris collector; (deposited in National Museum of Natural History [USNM], Washington, DC): **Arizona**, Cochise Co. Chiricahua Mts., East Turkey Creek, 6400’, 13.viii.1967 (2f), J. G. Franclemont; Onion Saddle, 7600’, 6.vii–21.viii.1966 (9m, 3f), 6.vii–21.viii.1967 (4m, 4f), J. G. Franclemont; Pima Co., Madera Canyon, Santa Rita Mts., 5900’, 11–16.ix.1950 (4f), R. H.Reid/C. W. Kirkwood; 30.vii–29.ix.1959 (12m, 30f), R. W.Hodges.

#### Etymology.

The name *arizonensis* (adjective) denotes the geographic locality from which the species is described.

#### Diagnosis.

Antaeotricha arizonensis immediately separates from other Antaeotricha species by its narrow elongate medium gray forewings with patchy dark maculation and unmarked fuscous hindwings.

#### Description.

Sexes similar except antenna and genitalia. Forewing length 9–11 mm (n = 25). **Adult** (Figs. 1–3): *Head* - (Antenna grayish brown with a few scattered white scales on scape; heavily ciliated ventrally in males, not ciliated in females. Frons with brown and white scales; labial palpus upcurved extending beyond crown of head, speckled brown and white with basal segment interiorly white. *Thorax* – Grayish-brown speckled with small white scales, tegula speckled brown and white. *Legs* – Speckled brown and white dorsally, white ventrally. Abdomen – Dorsally ochreous fuscous, whitish ventrally in ungreased specimens. *Wings* – Forewing clothed with a mixture of white, gray, and grayish-brown scales producing an apparent medium gray ground color. Dorsal forewing dark maculation consists of: black basal patch with zigzag distal margin; narrow antemedial black band, convex from costa to a mid-wing pale broken segment, then inwardly angulate from a dark longitudinal spot to junction at inner margin from which an irregular narrow band extends diagonally upward and outward to a discal apex black spot; a narrow post-medial black band extends basally from the costa to just above the discal apex black spot from which it forms a convex semicircle terminating at the inner margin; the terminal line consists of eight black spots; a dark patch extends along the costa from the antemedian to postmedian bands over the upper one-third of the wing. Fringe fuscous. Dorsal hindwing fuscous, unmaculated, with fuscous fringe. Ventral surfaces fuscous, with forewing slightly darker than hindwing.The forewings of worn specimens may appear nearly uniformly gray (Fig. 3). *Male genitalia* (Figs. 4–7; 4 dissections by author, 3 slides from J. A. Powell, 1 slide from USNM) – *Uncus*: decurved, narrow basally with long spatulate apex; gnathos expanded at tip with straight margin, but upcurved at middle and outer edges; vinculum complete, with dorsally produced projecting frontal process that tapers to an apical point. *Anellus*: without distinct lobes. *Valva*: with thumb-like projection on costa bearing long recurved bifurcate setae; *Aedeagus*: long, robust; cornuti four robust spines, three of similar length, one shorter. *Female genitalia* (Figs. 8–9; 2 dissections by author, 3 prepared slides [USNM]) – Genital plate broad and long with tongue-like projection into ostium bursae; ostium bursae large, urn-like, opening into a large spherical membranous sac, from which the ductus bursae originates at one side; inception of ductus seminalis is just below ostium; ductus bursae membranous, initially narrow then expanding below inception of ductus seminalis into a long wide uniform-diameter tube; corpus bursae spherical with one large signum; signum a circular disk with central outward projection.

#### Distribution and biology.

Mountain ranges in Southeastern Arizona (Cochise, Pima cos.) and southwestern New Mexico (Grant Co.). Reared from Quercus hypoleucoides A. Camus at Madera Canyon and Miller Canyon. Adults from mid-June to October suggests more than one generation.

## Supplementary Material

XML Treatment for Antaeotricha arizonensis
